# Functionalized Reduced Graphene Oxide Thin Films for Ultrahigh CO_2_ Gas Sensing Performance at Room Temperature

**DOI:** 10.3390/nano11030623

**Published:** 2021-03-03

**Authors:** Monika Gupta, Huzein Fahmi Hawari, Pradeep Kumar, Zainal Arif Burhanudin, Nelson Tansu

**Affiliations:** 1 Department of Electrical and Electronic Engineering, Universiti Teknologi PETRONAS, Seri Iskandar 32610, Perak, Malaysia; pradeep.hitesh@gmail.com (P.K.); zainalarif.burhanudin@utp.edu.my (Z.A.B.); 2 Center of Nanostructures and Nanodevices (COINN), Universiti Teknologi PETRONAS, Seri Iskandar 32610, Perak, Malaysia; 3 School of Electrical and Electronic Engineering, The University of Adelaide, Adelaide, SA 5005, Australia; nelson.tansu@adelaide.edu.au; 4 Center for Photonics and Nanoelectronics, Department of Electrical and Computer Engineering, Lehigh University, 7 Asa Drive, Bethlehem, PA 18015, USA

**Keywords:** graphene, gas sensor, functionalization, oxygen functional groups, response time, recovery time, room temperature sensing

## Abstract

The demand for carbon dioxide (CO_2_) gas detection is increasing nowadays. However, its fast detection at room temperature (RT) is a major challenge. Graphene is found to be the most promising sensing material for RT detection, owing to its high surface area and electrical conductivity. In this work, we report a highly edge functionalized chemically synthesized reduced graphene oxide (rGO) thin films to achieve fast sensing response for CO_2_ gas at room temperature. The high amount of edge functional groups is prominent for the sorption of CO_2_ molecules. Initially, rGO is synthesized by reduction of GO using ascorbic acid (AA) as a reducing agent. Three different concentrations of rGO are prepared using three AA concentrations (25, 50, and 100 mg) to optimize the material properties such as functional groups and conductivity. Thin films of three different AA reduced rGO suspensions (AArGO25, AArGO50, AArGO100) are developed and later analyzed using standard FTIR, XRD, Raman, XPS, TEM, SEM, and four-point probe measurement techniques. We find that the highest edge functionality is achieved by the AArGO25 sample with a conductivity of ~1389 S/cm. The functionalized AArGO25 gas sensor shows recordable high sensing properties (response and recovery time) with good repeatability for CO_2_ at room temperature at 500 ppm and 50 ppm. Short response and recovery time of ~26 s and ~10 s, respectively, are achieved for 500 ppm CO_2_ gas with the sensitivity of ~50 Hz/µg. We believe that a highly functionalized AArGO CO_2_ gas sensor could be applicable for enhanced oil recovery, industrial and domestic safety applications.

## 1. Introduction

The need for high-quality gas sensor development is increasing for the detection of various environmental pollutants that have an adverse effect on humans, animals, and plants. Over the past few years, atmospheric pollutants like CO_2_ are being produced as a continuous growth of industries, deforestation, and burning of fossil fuels. Furthermore, CO_2_ is one of the main causes of global warming and climate change. Moreover, CO_2_ gas is also attracting the serious attention of researchers in various fields such as CO_2_ storage, capture, and utilization. The level of CO_2_ in the atmosphere is unacceptably increasing day by day [1]. Therefore, practical and compact CO_2_ gas sensors demand to be explored critically. Numerous sensors on different platforms such as interdigitated electrodes (IDE) [2], quartz-crystal microbalance (QCM), and field-effect transistor (FET) [3] have been explored for the detection of CO_2_ gas. QCM is a sensitive piezoelectric resonator and its features such as high sensitivity, good reversibility, fast response, accuracy, robustness, availability, affordability, and simple integration with other electronic components, make QCM a preferred sensing platform than IDE and FET for CO_2_ gas detection [4,5,6]. The response of QCM-based gas sensors greatly depends on the sensing material coated on their electrodes. Various pristine and hybrid materials, such as SnO_2_ [7], Au-La_2_O_3_ doped SnO_2_ [8], ZnO [9], ZnO-CuO [10], BaTiO_3_–CuO [11], and molybdenum-tungsten oxide [12] materials, have been investigated as sensing material for the detection of CO_2_ gas. However, these materials require a very high temperature (200–600 °C) during gas sensing.

Novel materials, such as amino-ZnO [13], Ru@WS_2_ [14], vanadium oxide [15], nanodiamonds [16], Al/maPsi/n-Si/Al [17], and carbon nanotube [18], have been recently explored for CO_2_ gas sensing at room temperature. However, the sensing thin films of these materials require a mild thermal treatment before the sensing at room temperature. In addition, the gas sensors based on these materials have realized the longer recovery time (2–6 min). Besides these materials, graphene and its hybrids [15,19,20,21] are emerging as the promising contender for CO_2_ detection at room temperature due to their extraordinary properties such as high surface to volume ratio, high conductivity, and high chemical reactivity [22]. Owing to its very high volume–exposure ratio, a small amount of the graphene-based material enriched with the high surface area can provide ample active sites for high adsorption of the analyte gas molecules [23,24]. The derivative of graphene—reduced graphene oxide (rGO)—has also attracted much research attention in various application including catalysts [25], bio/gas sensors [26,27], electronic devices, and transparent electrodes [28], due to its facile implementation and outstanding properties such as hydrophilic nature, low-cost production, tunable optical band gap, and large surface area with high catalytic reactivity. 

Various methods have been investigated to reduce the GO for CO_2_ gas detection at room temperature, such as thermal reduction [15], exfoliation [29], spray pyrolysis technique [30], and the hydrogen plasma technique [31]. However, these methods need a high temperature for the reduction of GO and its mass production is limited. On the contrary, the chemically reduced graphene requires low temperature for the reduction of GO and it is suitable for mass production [32]. Moreover, the chemical reduction of GO offers the ease of surface modification and the functionalization of graphene oxide. The GO contains several oxygen functional groups (OFGs), such as the carboxyls, carbonyls, epoxides, and hydroxyls, which are covalently or non-covalently bonded in the form of hydrogen bonds and π-π bonds on its edge and basal plane [33,34]. The OFGs at the rGO surface is the dominant factor for analyte gas molecule adsorption at room temperature. These OFGs cause disruption to the π–π network that manifests as defects in the graphene sheet [35,36]. The π–π conjugate network of sp^2^ hybridized carbon structure and the sheet conductivity are restored when the OFGs are released from the GO sheet during the reduction process [37]. The OFGs can be efficiently tailored by the chemical reduction method.

During the reduction of GO, the key interests are (i) the fraction of OFGs are involved during the reduction process, (ii) how many functional groups are leaving the surface, (iii) how many functional groups remain at the edge plane, and basal plane, and (iv) the formation of defects on the edge or basal plane when the OFGs leave the GO surface. Although both edge and basal plane OFGs take part in the molecule adsorption, the edge plane OFGs dominates. The basal plane OFGs (hydroxyl) work as the surface trap sites for the charge carriers (electrons and holes) [38]. Due to the basal plane trap sites, high-temperature annealing is required to achieve the desorption of gas molecules [39]. For the fast CO_2_ gas detection at room temperature, the edge functionalities are favorable as compared to the basal plane functionalities. The amount of OFGs at the edge plane and basal plane can be modified by changing the synthesis parameters such as synthesis duration, temperature, the type and concentration of the reducing agent [40]. Among these synthesis parameters, the concentration of the reducing agent has a strong effect on OFGs and CO_2_ gas sensing performance, which has not been systematically investigated.

In this work, we have thoroughly investigated the effect of reducing agent concentration on the OFGs and the corresponding effect on the CO_2_ gas sensing (response and recovery time). The number of OFGs at the rGO surface is highly sensitive towards the reducing agent (ascorbic acid) concentration. The edge plane functional groups are the dominant reactive sites for the adsorption of the analyte gas molecules. A green eco-friendly and non-toxic reducing agent, ascorbic acid (AA) was used for reducing the GO at low temperature. The typical reducing agents such as hydrazine (N_2_H_4_) and sodium borohydride (NaBH_4_) are toxic and not environmentally benign [40,41]. AA is an organic compound with antioxidant properties and its high solubility in the water offers a mildly acidic solution [40]. Unlike typical reducing agents, AA facilitates the high removal of basal functional groups from the GO surface [42,43]. Three concentrations (25 mg, 50 mg, 100 mg) of AA were investigated to achieve a highly edge functionalized graphene. The characterization results revealed that the AA reduced GO (AArGO) has wrinkles, defects, and a high amount of OFGs at the edges. Later, we evaluated the sensing characteristics of AArGO materials for CO_2_ gas at room temperature. It was observed that the AArGO material with the lowest concentration has the highest edge functional groups and showed the best sensing performance towards CO_2_ gas.

## 2. Materials and Methods

### 2.1. Materials and Reagents

Graphene oxide (GO) paste (95%) was purchased from Graphenea (San Sebastian, Spain). Ascorbic acid, ethanol (95%), and acetone (95%) were purchased by Sigma Aldrich, St. Louis, MO, USA. All the chemicals were of analytical grade, no further purification was required for conducting experiments. Deionized water was used in all preparations.

### 2.2. Synthesis of Reduced Graphene Oxide and Thin-Film Development

**Figure 1:**
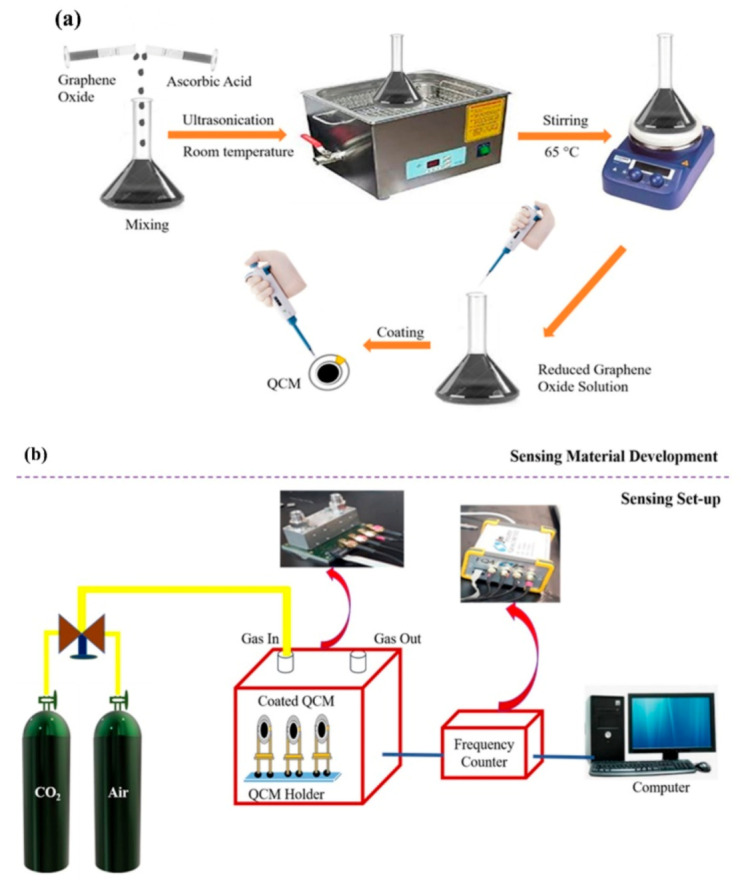
Schematic illustrations for (**a**) material development and (**b**) AArGO-coated quartz crystal microbalance (QCM) based CO_2_ gas sensing set-up.

To prepare the reduced-graphene oxide (rGO), an aqueous suspension of GO in de-ionized (DI) water was initially prepared followed by ultrasonication for 15 min to obtain a uniform aqueous dispersion. Then, the GO suspensions with three different AA concentrations (25 mg, 50 mg, and 100 mg) were prepared. The suspensions were prepared by slowly and carefully adding the ascorbic acid into aqueous GO. The prepared mixture suspensions were then vigorously stirred on the hot plate for 1 h at 65 °C under room environment conditions. The color of the GO solution was gradually changed from brown color to blank color signifying the reduction of GO into rGO. After the reduction, the corresponding synthesized rGO suspensions were marked as AArGO25, AArGO50, AArGO100 for respective AA concentrations. The schematic illustration of material preparation is shown in Figure 1a.

The thin films of synthesized AArGO suspensions were developed by drop-casting technique. Prior to thin-film development, the target substrates such as glass and SiO_2_ (300 nm)/Si were washed in acetone, isopropyl alcohol, DI water, and ethanol solvents using ultrasonication for 15 min in each solvent.

### 2.3. Structural, Elemental, Morphological, and Electrical Characterizations

The functional groups of the synthesized materials were investigated by Fourier Transform Infrared (FTIR) spectroscopy (Bruker Instruments, model Aquinox 55, Stuttgart, Germany). The crystal structure of the material samples was examined by X-ray diffraction (XRD) measurements for the scanning range 2–80° (X’Pert3 Powder) and Raman spectroscopy (Horiba Jobin Yvon HR800, Yvon, France) at ambient temperature with 514 nm laser excitation for 200 to 4000 cm^−1^ spectrum regions. The surface morphology of the samples was investigated using scanning electron microscopy (SEM, Leo 1430vp, Carl Zeiss AG, Jena, Germany) and transmission electron microscopy (TEM, Zeiss Libra 200FE, Jena, Germany), respectively. The presence of essential elements on the material surface was analyzed by X-ray photoelectron spectroscopy (XPS, Thermo Scientific, Sunnyvale, CA, USA) with a monochromatic A1 Kα1 source (photon energy 1596 eV). The electrical properties such as conductivity, carrier concentration, and sheet resistance of thin films of prepared materials were measured using a four-point probe system (Lucas Lab 302) with Keithley 2400 source meter. The thin films of all materials were prepared onto the glass and SiO_2_ substrates.

### 2.4. Sensor Fabrication and Device Performance

The silver (Ag) coated 10 MHz quartz crystal microbalance (QCM, WTL International China, Shenzhen, China) was used as a sensing platform. QCM resonators were cleaned in acetone, isopropyl alcohol, DI water, and ethanol solvents using ultrasonication for 15 min in each solvent. A drop-casting technique was used to prepare the thin films of the sensing material. 10 μL of GO and AArGO suspensions were taken using a micropipette to drop-cast the QCM substrates on both sides under identical conditions. The material coated QCMs were then dried at room temperature.

An uncoated QCM is tested for CO_2_ gas and no response is observed for the same. The gas sensing experiments were performed by using a custom-made gas sensing setup at room temperature. Figure 1b illustrates the schematic of the QCM-based gas sensing experimental setup. The AArGO coated QCM resonators were placed in a QCM holder. Then, the QCM holder was connected to a frequency counter (FQ4, JLM Innovation, Tubingen, Germany). The output of the frequency counter was given to a compatible computer via an RS–232 serial communication port to acquire the sensing characteristics (response time and recovery time). Initially, the air as the carrier gas was purged to AArGO-coated QCM for 60 s. Air was then switched off and instantaneously CO_2_ gas (with 500 ppm or 50 ppm concentration) was purged on the AArGO-coated QCM surface. Meanwhile, the air was again purged to desorb the CO_2_ gas molecule. The QCM frequency modulates with the adsorption/desorption of CO_2_ molecules. The frequency shift varied rapidly, and the frequency counter was used to record the frequency shifts of the QCM sensor. The sensitivity of AArGO coated QCM sensors were calculated by the ratio of frequency shift/coating amount (Hz/µg) to compare the performance of AArGO25, AArGO50, and AArGO100 coated QCM sensors. The response and recovery times of the sensor were defined as the times needed for the sensor to achieve 90% and 10% of the maximum shifts during the sensing and purging processes, respectively.

## 3. Results and Discussion

### 3.1. FTIR Analysis

**Figure 2:**
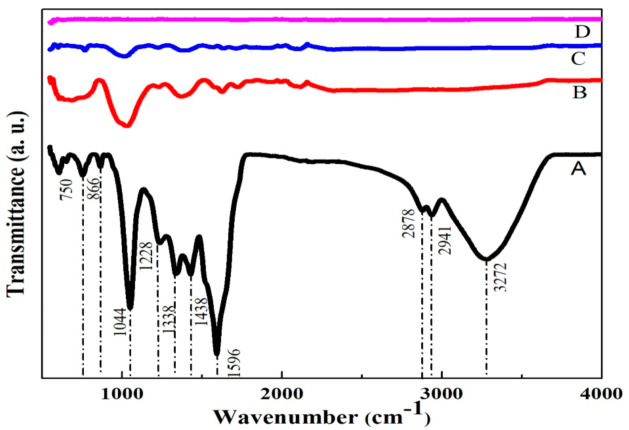
FTIR spectra of GO (**A**), AArGO25 (**B**), AArGO50 (**C**), and AArGO100 (**D**).

The structural properties of GO and rGO samples have been studied by using FTIR spectroscopy. Figure 2 shows the FTIR spectra of GO (A), AArGO25 (B), AArGO50 (C), and AArGO100 (D). The essential absorption peaks are tabulated in Table 1. The GO spectrum shows a broad absorption band centered at 3182 cm^−1^ attributed to the –OH stretching vibration and indicating the presence of –OH and –COOH functional groups within the structure. This band is related to the absorbed and inhibited water molecule to atmospheric moisture. The peaks at 1724, and 1620 cm^−1^ were observed suggesting the vibrations of C=O stretching, and C=C alkene group stretching. Some other peaks at 1225 and 1044 cm^−1^ were also observed indicating the C–O stretching of epoxy groups and C–O stretching vibration of an alkoxy group, respectively. In the AArGO spectra, it was noticed that the peak intensity of most of the oxygens functional group (OFGs) was found to be decreased that indicates the successful reduction of graphene oxide by AA reducing agent. It was also observed that the peaks due to the hydroxyl group were absent ~3182 cm^−1^ in all the AArGO samples but the emergence of the absorption peak at ~1580 cm^−1^ (the aromatic structure) indicates the successful deoxygenation of the rGOs by AA [44].

**Figure 3:**
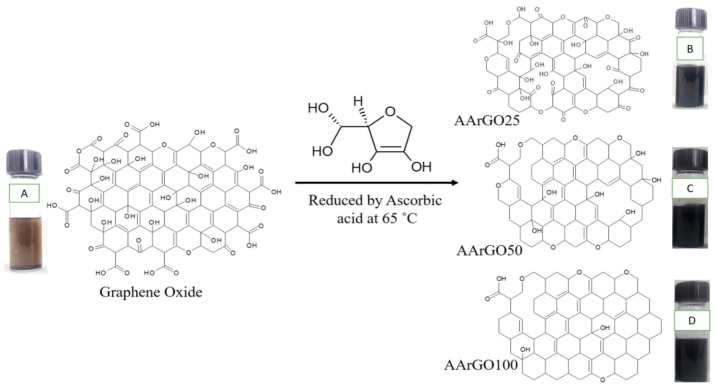
Representation of oxygen functional groups (OFGs) after the reduction of graphene oxide using ascorbic acid. Graphene oxide (**A**), AArGO25 (**B**), AArGO50 (**C**), AArGO100 (**D**).

Figure 3 depicts a representation of the reduction process of GO using AA and the formation of AArGO materials with different amounts of OFGs. In graphene oxide, the OFGs are attached between the graphene layers, at the edges, and the surface of graphene. When GO is chemically reduced, the AA interacts with these OFGs. AA reduces mainly the basal plane functional groups such as hydroxyls and epoxides [40,42]. The OFGs such as hydroxyls and epoxides are mainly fastened at the basal plane whereas the other OFGs such as carboxyls and carbonyls are dominant functional groups at the edges [34]. The existence of the carbonyl and carboxyl edge functionalities assures the presence of more active sites for the sorption of the analyte gas like CO_2_**.** However, in the case of GO (before the reduction), these OFGs do not behave as active sites because these covalently bonded OFGs are hard to detach from the GO surface at room temperature until an external excitation like thermal treatment is provided [45]. Furthermore, these edge functionalities majorly contribute to the high stability and low tendency of agglomerations in the AArGO [46]. The dispersion of edge functionalized graphene is better than basal plane functionalized graphene. Carbonyl and carboxyl OFGs contribute to better dispersion properties of the material [40]. The dispersion of sample AArGO25 was remained stable even up to one month (see Figure S1), indicating the AArGO25 sample has more carboxyl and carbonyl OFGs in comparison to the AArGO50 and AArGO100 samples.

The FTIR spectrum of AArGO25 showed a dominant peak at 1027 cm^−1^ attributed to the C–OH vibrational shift at edge located hydroxyl group [44]. This C–OH edge deformation peak is also favorable for sorption. Furthermore, the stretching vibration C=C peak was found to be shifted to 1580 cm^−1^ from 1596 cm^−1^. The residual epoxy groups form the carboxyl groups (C=O) with dehydroascorbic acid, indicated by a peak at 1731 cm^−1^. In AArGO50 and AArGO100, most of the OFGs are decomposed due to the high possibility of protonation. However, a small number of carboxyl groups still exist in AArGO50 and AArGO100 indicated by the presence of peaks at 1713 cm^−1^, 1724 cm^−1^ respectively [33].

### 3.2. XRD Analysis

**Figure 4:**
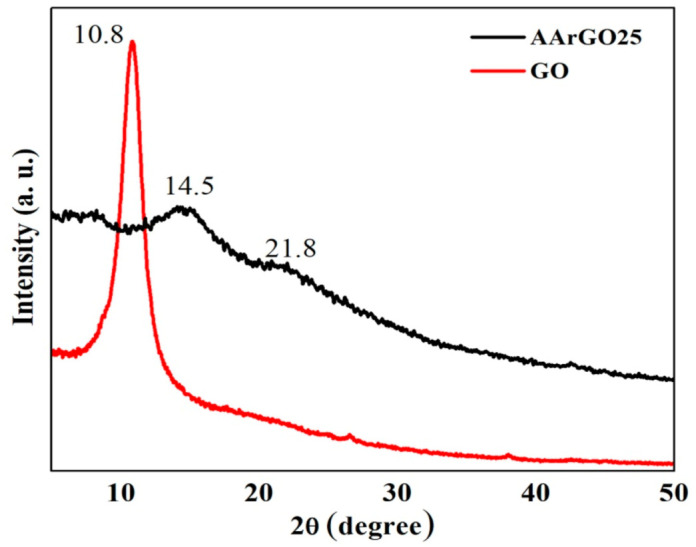
XRD patterns of GO and reduced graphene oxide (AArGO25).

The crystalline structure of GO and AArGO samples were investigated using the X-ray diffraction (XRD) patterns to verify the variation in OFGs, as shown in Figure 4. The various physical parameters were calculated from XRD and tabulated in Table S1. The characteristic (001) reflection peak for GO was observed at 2*θ* = 10.8° and the corresponding interlayer spacing (*d*) of 8.18 Å was determined using Bragg’s equation [33]. The characteristic (001) peak for the optimized rGO sample (AArGO25) was observed at 2*θ* = 14.5° position, close to the GO characteristic peak (2*θ* = 10.8°). Another weak but broader characteristic (200) reflection peak was also observed at 2*θ* = 21.8°. The *d* was determined to be 4.07 Å for AArGO25. The lower value of *d* for AArGO25 suggests the reduction of OFGs between the graphene layers. This indicates the successful reduction of GO and restoration of π-conjugate structure [44,47]. On the contrary, the higher *d* value of GO shows the negligible stacking of layers due to electrostatic repulsion between the layers [48].

The average values of crystallite width (*D*) [37] and in-plane crystallite size (*L*) [49] of AArGO25 were found to be 5.9 Å and 12.25 Å, respectively whereas, *D* and *L* were found to be 96 Å and 199 Å, respectively for GO. The lower values of *D* and *L* of AArGO25 attribute to the disruption of stacking order of graphite flakes and formation of defects caused by the repulsion of graphene layers [31]. These deformations and defects can be identified in the form of wrinkles which suggests the enhancement in the exposed surface area favorable for high analyte gas adsorption [40]. The average number of graphene layers per domain (*N*) was found to be 4 and 12 for AArGO25 and GO, respectively, indicating the shrinkage of graphitic domains. The agreement between the experimental and theoretical agreements shows a strong support on the number of the graphene layers per domain in the AArGO material.

### 3.3. Raman Analysis

**Figure 5:**
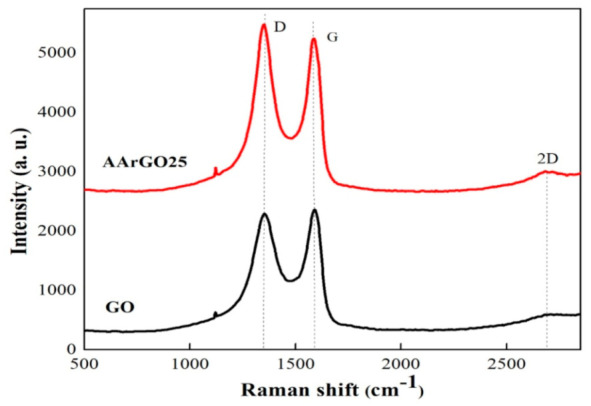
The Raman spectra of GO and AArGO25 thin films.

To verify the quality of GO and rGO, we have performed Raman spectroscopy of their thin films as presented in Figure 5. The major Raman features are tabulated in Table 2. The spectra of GO exhibited the D- and G- peaks at ~1352 cm^−1^ and ~1592 cm^−1^, respectively. The D- and G- peak both were downshifted compared to GO, indicates the reduction of GO by ascorbic acid. For AArGO25 thin film, D-peak is observed at ~1350 cm^−1^. The D-peak is originated due to sp^2^ spatial domain breathing modes and it is activated due to a defect. The higher intensity and narrow D-peak attributes to the defect and disorder of AArGO thin film but not dependent on the number of graphene sheets [50]. The G-peak was observed to be at ~1587 cm^−1^, the downshifting in the G-peak of the AArGO25 is attributed to the electron-donating behavior of AA that indicates the restoration of the π-conjugated structure of graphene. Therefore, the G-peak corresponds to C=C stretching vibrations and D-peak attributes disorder-induced mode [33]. The *I_D_/I_G_* intensity ratio was found to be 1.1 for AArGO25. The higher *I_D_/I_G_* intensity ratio of AArGO25 in comparison to the GO (*I_D_/I_G_* = 0.9) reflects some defects and the formation of a new graphitic domain (smaller spatial dimensions) [28,37,51]. The 2D peaks for GO and AArGO were noticed at ~2683 cm^−1^, and ~2701 cm^−1^, respectively. The corresponding *I_2D_/I_G_* intensity ratios were found to be 0.25 and 0.21, for GO and AArGO25 respectively, indicating the good electronic properties are maintained after the reduction (0.2 < *I_2D_/I_G_ <* 0.4) [50]. The low-intensity ratio of *I_2D_/I_G_* for AArGO25 also suggests that the prepared sample has few-layer graphene (4–6 layers, *I_2D_/I_G_* < 0.5) [52], showing an agreement with XRD-based calculation of the number of graphene layers.

### 3.4. Surface Morphology

**Figure 6:**
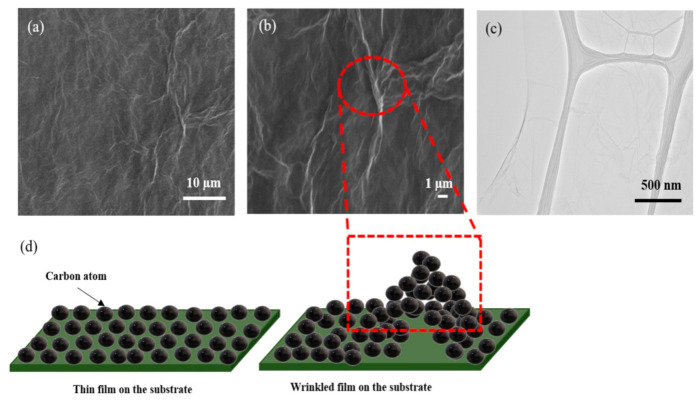
SEM of AArGO25 at (**a**) lower magnification and, (**b**) higher magnification, (**c**) TEM image, (**d**) Schematic illustration of wrinkles on AArGO25 surface.

To investigate the morphology of the rGO sample, the SEM and TEM characterizations were carried out. The morphology of AArGO25 investigated using SEM and TEM is shown in Figure 6. The AArGO25 was found to have a pleated surface as shown in the SEM image (Figure 6a). Several corrugations and wrinkles were also observed on the high magnification SEM image of the surface as presented in Figure 6b. Figure 6c illustrates the AArGO25 surface on the holey copper grid during TEM imaging. The surface was found to be a continuous thin layer. Some folds and wrinkles were also identified on edge of the surface. These corrugations on the AArGO25 surface can modify its electronic structure, mechanical, optical, and chemical properties [22,27].

The formation and the effect of wrinkles on the AArGO surface are illustrated in Figure 6d. The wrinkles are formed due to the interaction between substrate and graphene mainly caused by the difference between Young’s moduli of substrate and graphene [53,54]. The wrinkled sheets suggest an increased surface area of AArGO25 thin film that may be helpful to enhance the adsorption of the gas molecule which is consistent with what had been reported in the literature [40].

### 3.5. XPS Analysis

**Figure 7:**
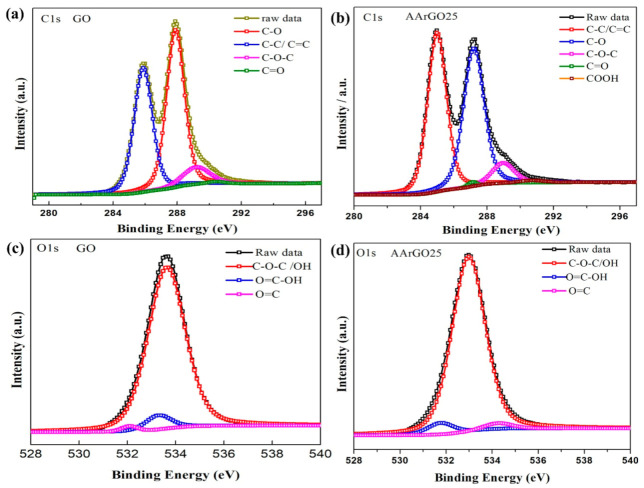
C1s and O1s XPS spectra of GO and AArGO25 thin films. C1s spectra of (**a**) GO and (**b**) AArGO25. O1s spectra of (**c**) GO and (**d**) AArGO25.

The chemical composition and elemental analysis for the GO and AArGO25 were investigated using XPS-C1s spectra as illustrated in Figure 7. The binding energy profile of deconvoluted C1s and O1s spectra are tabulated in Table 3, respectively. After deconvolution, the C1s spectrum of GO showed four major peaks that correspond to C–C/C=C (285.8 eV, ~27.9%) in aromatic rings, C–O epoxy and alkoxy (287.9 eV, ~34.3%), C–O–C (289.21 eV, ~5.75%), and C=O double bond (290.4 eV, ~0.53%) as depicted in Figure 7a. The reduction of GO mainly affected the C–C/C=C bond and the intensity of C–C/C=C peak was dramatically increased from 27.9% (GO) to 33.35% in AArGO25 as illustrated in Figure 7b, indicating a high removal rate of the epoxide and hydroxyl (C–O) groups and abundance of edge functionalities. These changes in OFGs lead to the enhanced dispersion stability of AArGO25 material [46]. A similar observation on the reduction of hydroxides was found during the FTIR analysis of AArGO25. The peak intensity of the AArGO25 carbonyl group (C=O) was found to be higher than that of GO, attributing to a higher number of carbonyl elements of the AArGO25 material. These carbonyl OFGs form the defective surface of AArGO25 [22]. These defects are the main active sites for the adsorption of the CO_2_ gas molecules. In addition, the C/O ratio was also slightly increased from 2.17 (GO) to 2.44 (AArGO25), reflecting the reduction of GO with an abundance of OFGs. Additionally, FWHM (full width at half maximum) value was found to be 1.52 and 1.33 for GO and AArGO25, respectively. The smaller FWHM value for AArGO25 suggests its good electrical conductivity with a large number of holes [40]. These holes are formed by the breaking of C–C bonds in the basal plane and interactions between the neighboring hydroxyl and epoxy groups during the reduction process [22,55].

Moreover, the analysis of the high-resolution O1s spectra was performed to get considerable information on sorption. The O1s spectra of GO and AArGO25 are shown in Figure 7c,d, respectively. The O1s spectrum of GO decomposed in three peaks associated with C–O (~533.6 eV), C=O (~533.0 eV), and O–C=OH (~533.2 eV) as shown in Figure 7c, whereas these three major peaks for AArGO25 were found to be ~534.9 eV, ~534. 2 eV, and ~531.7 eV, respectively. The observed significant reduction in the binding energy of the C–O group for AArGO25 is found to be agreed with the trend observed by Chen et al. [40], Rabchinskii et al. [44]. Furthermore, the amount of oxygen in AArGO25 was reduced from 31.47% (GO) to 29.08%, indicating the reduction of GO.

### 3.6. Electrical Analysis

**Figure 8:**
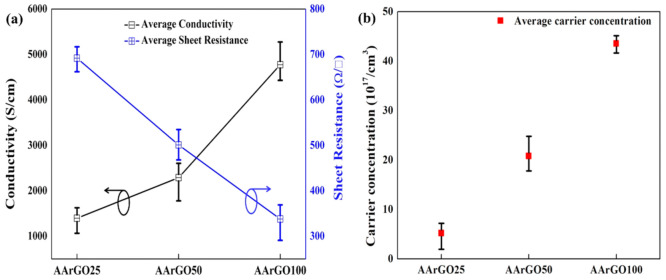
The electrical properties of AArGO thin films: (**a**) conductivity and sheet resistance, (**b**) carrier concentration.

The electrical properties of graphene can be tailored by varying the amount of OFGs on the graphene surface. Based on the abovementioned FTIR and XPS results, AArGO25 has a high amount of OFGs. These OFGs act as the scattering centers which results in lower electrical conductivity or high sheet resistance [56]. With the increase of AA concentration, the amount of OFGs was gradually reduced from AArGO25 to AArGO100. The room temperature average conductivity (*σ*) and sheet resistance (*R_S_*) for AArGO25 thin film was measured to be 1389 S/cm and 691 Ω/□, respectively, indicating the existence of a high amount of OFGs. For AArGO50 and AArGO100 thin films, the average *σ* of 2295 S/cm and 4790 S/cm was measured as shown in Figure 8a. Besides, the average *R_S_* for them was found to be 508 Ω/□ and 331 Ω/□, respectively. Similar results were also reported for rGO thin films by Savchak et al. [57], Ren et al. [58].

As a comparison, the measurements conducted on the GO-thin films prepared under room-temperature exhibited insulating nature. The AArGO25 thin film has comparatively high sheet resistance. This *R_S_* instigates from the attraction/repulsion force between the graphene sheets (stacking), causing defects in the AArGO25 thin films, that oppose the charge carrier transfer [59]. The OFGs such as epoxy, carboxyl, and hydroxyl groups on both sides of rGO sheets may contribute to imperfect surface morphology [60]. The reduction of GO by smaller AA concentrations (i.e., 25 mg) leads to a higher number of OFGs that correspondingly lowers its (AArGO25 thin films) *σ* and increases the *R_S_*. The higher degree of reduction leads to a lower amount of OFGs on the surface of thin film and consequently, increases the carrier concentration [56]. The average carrier concentration was measured to be 5.205 × 10^17^/cm^3^, 2.08 × 10^18^/cm^3^, and 4.355 × 10^18^/cm^3^ for AArGO25, AArGO50, and AArGO100, respectively as presented in Figure 8b. The carrier concentration of the AArGO50 and AArGO100 thin films were found to be increased attributing to the reduction of OFGs upon the increment in the amount of AA reducing agent.

### 3.7. Gas Sensing

**Figure 9:**
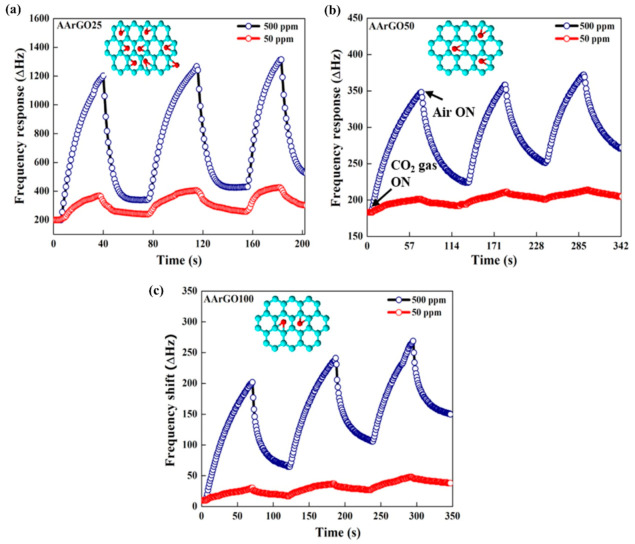
The response of CO_2_ gas sensors with AArGO-based sensing thin films. Absorption/desorption curve of (**a**) AArGO25, (**b**) AArGO50, and (**c**) AArGO100. Insets illustrate the variations in the number of OFGs (in red) for different AArGO.

The sensing properties such as response time and recovery time of CO_2_ gas sensors at 500 ppm and 50 ppm with different AArGO (sensing material) thin films are presented in Figure 9. All the AArGO-coated QCM sensors were observed to have a significant, continuous, and repeatable response for CO_2_ gas (500 ppm or 50 ppm in N_2_) at room temperature (RT). The stable response of sensors indicates the robustness of the coated sensing material. Among three gas sensors, the best response for CO_2_ gas was shown by the AArGO25-based sensor at 500 ppm and 50 ppm. Its response time (*T_res_*)/recovery time (*T_rec_*) was measured to be ~26/10 s for 500 ppm and ~25/18 s for 50 ppm as shown in Figure 9a. For AArGO50 and AArGO100-based sensors, the *T_res_/T_rec_* was measured to be ~40/39 s, ~52/30 s for 500 ppm CO_2_ and ~46/47 s, ~58/35 s for 50 ppm CO_2_, respectively as depicted in Figure 9b,c, respectively attributing to the small number of carbonyl-OFGs (illustrated in the insets) presented on the thin film surface of AArGO50 and AArGO100 sensors. The low desorption was due to the π–π interaction between the adsorbate (CO_2_ gas molecule) and adsorbent (AArGO surface) [60]. The response and recovery time comparison of the AArGO25 sensor is tabulated in Table 4. The AArGO sensor is found to be better as compared to those reported in the published literature on CO_2_ sensor at room temperature.

Although all three samples show a good response towards CO_2_ gas, the adsorbed CO_2_ molecules do not desorb completely during the recovery process revealing drift to the baseline. The AArGO25-coated QCM sensor almost achieves baseline after each adsorption/desorption cycle because the high amount of edge-OFGs in AArGO25 are the governing factor for CO_2_ adsorption/desorption. However, The response of the AArGO100-coated QCM sensor was found to be repeatable but showed the highest upward drift to baseline, which could be mainly attributed to the incomplete desorption of CO_2_ gas molecule [60]. It can be suggested that this incomplete desorption of CO_2_ molecules in the AArGO100-coated QCM sensor was due to higher hole carrier concentration. These excessive hole charge carriers are trap sites for CO_2_ gas molecules. The hole charge carrier concentrations gradually increase after each adsorption/desorption cycle [61] because the CO_2_ molecule is a strong acceptor, and it leaves a hole at the AArGO surface after desorption. The increased hole concentration reduces the CO_2_ molecule adsorption capacity gradually which results in baseline upward drift in the sensor response [15,55].

The GO-coated QCM sensor was also exposed to CO_2_ gas. The GO-based gas sensor, which was prepared by a room-temperature drying method, did not show any response upon the CO_2_ exposure possibly due to the unavailability of active sites. The surface properties of GO depend on the oxidation degree and our GO was found to be functionalized with mainly hydroxyl and epoxide groups [27,46]. Due to the oxidation during the GO synthesis, it loses its sp^2^ hybridization, making GO insulating [48,58,62]. Furthermore, these hydroxyls and epoxides are covalently bonded with carbon (C) atoms. These covalently bonded OFGs are hard to detach from the GO surface at room temperature. High-temperature annealing or other forces are required to detach them from the surface of GO [45].

**Figure 10:**
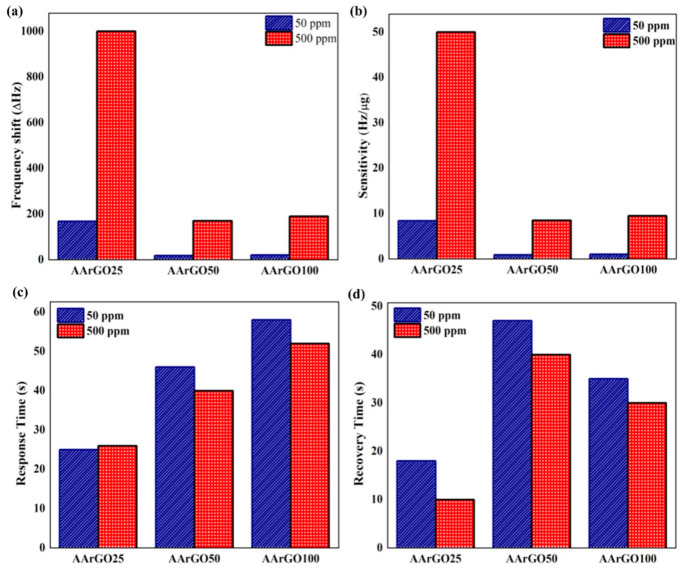
Comparisons of (**a**) frequency shift, (**b**) sensitivity, (**c**) response time, and (**d**) recovery time, of CO_2_ gas sensors with different AArGO sensing thin films.

A comparative study of sensing properties is depicted in Figure 10. Figure 10a shows the frequency shift (Δ*f*) variations for different CO_2_ gas sensors for 500 ppm and 50 ppm. The Δ*f* for AArGO25, AArGO50, and AArGO100 sensors was measured to be 1000 Hz, 170 Hz, 190 Hz for 500 ppm and 168 Hz, 18 Hz, 20 Hz for 50 ppm, respectively. The sensitivity of the QCM sensor depends on the coating amount of sensing material (AArGO). The sensitivity for AArGO25, AArGO50, and AArGO100 sensors was found to be 50 Hz/µg, 8.5 Hz/µg, 9.5 Hz/µg for 500 ppm and 8.4 Hz/µg, 0.9 Hz/µg, 1 Hz/µg for 50 ppm, respectively as shown in Figure 10b, demonstrating that the AArGO25-based gas sensor is a highly sensitive CO_2_ gas sensor. Figure 10c,d present a relative comparison of *T_res_*/*T_rec_* for all AArGO-based CO_2_ gas sensors for 500 ppm and 50 ppm. The AArGO25 showed the lowest *T_res_*/*T_rec_* attributing to a higher amount of OFGs. Furthermore, the AArGO25 exhibited the lowest conductivity, possibly due to the large amount of OFGs. Previous studies [56,63] also showed that the material with lower conductivity realizes the more sensitive response, which is also found to agree with this study.

**Figure 11:**
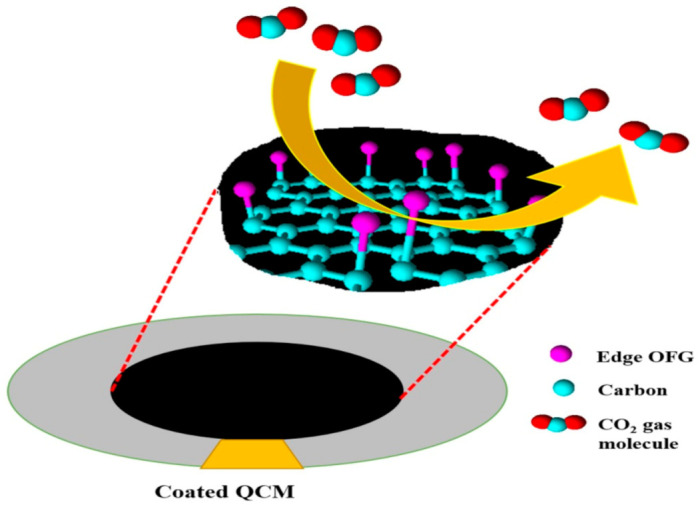
Schematic illustration of CO_2_ gas molecule adsorption on the surface of AArGO sheet. The edge plane carbonyls and edge-hydroxyls (marked in pink) bonded to the central carbon atom support π–π interactions between these OFGs and CO_2_ gas molecules.

The possible mechanism of the CO_2_ gas sensing by AArGO-coated QCM sensor is illustrated in Figure 11. When the air is purged on the AArGO coated QCM sensor at room temperature, the oxygen molecules are adsorbed on the AArGO surface by physisorption process due to Van der Waals and dipole interactions [4]. The adsorbed oxygen molecules are then ionized to oxygen ions $O_{2}^{-}$ by trapping the free electrons of the AArGO surface and relatively the oxygen concentration is increased at the surface [20]. The reaction process can be given as:

(1) $$O_{2}\left(\mathrm{gas}\right)\;↔\;O_{2}\left(\mathrm{ads}\right)$$

(2) $$O_{2}\left(\mathrm{ads}\right)\;+\;e^{-}\;↔\;O_{2}^{-}\left(\mathrm{ads}\right)$$

When the AArGO-coated QCM sensor is exposed to oxidizing CO_2_ gas, the layered oxygen ions interact with the CO_2_ molecule and form the carbonate ions [30,64,65].

(3) $${\mathrm{CO}}_{2}\left(\mathrm{gas}\right)\;↔\;{\mathrm{CO}}_{2}\left(\mathrm{ads}\right)$$

(4) $${\mathrm{CO}}_{2}\left(\mathrm{gas}\right)\;+\;e^{-}\;\to \;{\mathrm{CO}}_{2}{}^{-}$$

(5) $${2\mathrm{CO}}_{2}\left(\mathrm{gas}\right)\;+\;O_{2}^{-}\;+\;e^{-}\;\to \;{2\mathrm{CO}}_{3}^{-}\left(\mathrm{ads}\right)$$

Each CO_2_ molecule consumes an electron during its interaction with the AArGO surface and leaves a hole when detached. Therefore, the hole carrier concentration in AArGO material is increased thereby, the resistance (*R_S_*) of the AArGO film surface is reduced at room temperature [30].

## 4. Conclusions

In this work, we have successfully synthesized the highly edge functionalized rGO thin films as the sensing material for CO_2_ gas detection at room temperature. The AArGO-based sensing thin films were developed by drop-casting the suspensions on the Ag electrode of QCM sensors. The amount of OFGs at the AArGO surface was tuned by optimizing the ascorbic acid concentration. The standard characterization techniques exhibited that the AArGO25 thin film has the highest amount of OFGs, wrinkles, defects, and anti-agglomerating nature with electrical conductivity of ~1389 S/cm. The result revealed that the AArGO sample with the lowest AA concentration has the highest OFGs on its surface thereby exhibiting the highest response (1000 Hz) at 500 ppm CO_2_ gas. In addition, the highly edge functionalized AArGO25-based QCM gas sensor demonstrated the ultrafast sensing response with good repeatability and almost complete desorption. A short response time and recovery time of 26 s and 10 s, respectively, were observed for 500 ppm CO_2_ gas at room temperature with the sensitivity of ~50 Hz/µg. For 50 ppm, the AArGO25 sensor showed almost the same *T_res_* as for 500 ppm but have higher *T_rec_* (18 s). This study reveals that the rGO sensing material with a small amount of ascorbic acid (25 mg) is more efficient for CO_2_ gas sensing. These findings render the interesting possibilities of tuning the OFGs at the rGO surface which may be promising not only for the future gas sensors, but also for other potential applications such as catalysts, biosensors, lubrication, and electronic devices. The selectivity of the edge-functionalized rGO-based gas sensor will be studied in future work.

## Figures and Tables

**Table 1:** FTIR analysis of the GO and the AArGO samples.

Assignments	Absorption Frequencies (cm^−1^)			
	GO	AArGO25	AArGO50	AArGO100
O–H vibration	3272	-	-	-
C–H vibration	2941	2323	-	-
C=O stretching	1786	1731	1713	1724
aromatic C=C vibration	1596	1580	1565	-
–COOH stretching	-	1623	1630	1683
C–OH bending	1338	1370	1394	-
C–O–C bending	1228	1228	1224	-
C–O stretching	1044	1027	1015	1015

**Table 2:** The Raman fingerprints of GO and AArGO25 thin films.

Sample	D	G	2D	*I_D_*/*I_G_*	*I_2D_*/*I_G_*
GO	1352	1592	2683	0.9716	0.2512
AArGO25	1350	1587	2701	1.085	0.218

**Table 3:** C1s and O1s XPS Spectra of GO and AArGO25 thin films.

C1s XPS Spectra							
Sample	C–C/C=C (%)	C–O (%)	C–O–C (%)	C=O (%)	COOH (%)	C/O Ratio	FWHM of C1s
GO	27.92	34.33	5.75	0.53	-	2.17	1.52
AArGO25	33.35	31.58	4.99	0.44	0.56	2.44	1.33
**O1s XPS Spectra**							
**Sample**	**C–O–C (%)**		**C=O (%)**		**COOH (%)**	**FWHM** **of O1s**	
GO	29.23		0.44		1.8	1.29	
AArGO25	26.91		0.76		1.41	1.2	

**Table 4:** Performance comparison of room temperature CO_2_ gas sensing.

Materials	Synthesis/Fabrication Method	CO_2_ Concentration (ppm)	Response Time	Recovery Time	Reference
Amino−ZnO nanohybrids	in situ hydrothermal	500	206 s	354 s	[13]
Reduced graphene oxide	Hydrogen plasma reduction	1500	4 min	4 min	[31]
Self-standing MWCNTs/alumina composite film	Hydrolysis	50–450	53.7 s	14.15 s	[66]
Carbon nanotube on a polyimide substrate	Chemical vapor deposition	50	12 s	56 s	[18]
Ru-decorated WS_2_ quantum dots	Two-step synthesis process	500–5000	52 s	138 s	[14]
Cyano-terminated ethynylated-thiourea	Chemical synthesis	10–1000	1 min	3 min	[2]
Al/maPsi/n-Si/Al	Laser assisted etching (LAE)	-	2.9 min	4.1 min	[17]
Graphene oxide	Spray pyrolysis on fluorine tin oxide	-	125 s	110 s	[15]
Nanocrystalline diamond	Microwave Plasma CVD process	2500	120 s	400 s	[16]
Vanadium Oxide	Vacuum thermal evaporation	-	50 s	125 s	[65]
AArGO	Chemical reduction	500	26 s	10 s	This work

## Data Availability

The data are available upon request from the corresponding authors.

## Supplementary Materials

The following are available online at https://www.mdpi.com/2079-4991/11/3/623/s1, Figure S1. Aqueous dispersion of different AArGO: GO (A), AArGO25 (B), AArGO50 (C), and AArGO100 (D) after 1, 2, 5, 10, 20, and 30 days. Table S1. Physical parameters of GO and AArGO25 obtained from XRD.
