# Supplementary material for: Functionalized Reduced Graphene Oxide Thin Films for Ultrahigh CO2 Gas Sensing Performance at Room Temperature
Source: Nanomaterials (Basel). 2021 Mar 3;11(3):623. doi: 10.3390/nano11030623 (PMC7998141; doi:10.3390/nano11030623)
Supplement: Supplementary file 1 [file nanomaterials-11-00623-s001.pdf]

Supplementary Information

Functionalized Reduced Graphene Oxide Thin Films for Ultrahigh CO2 Gas Sensing Performance at Room Temperature

Monika Gupta [1,2]*, Huzein Fahmi Hawari [1,2]*, Pradeep Kumar [1,2], Zainal Arif Burhanudin [1,2] and Nelson Tansu[3,4]

[1] Department of Electrical and Electronic Engineering, Universiti Teknologi PETRONAS, Seri Iskandar 32610, Perak, Malaysia; pradeep.hitesh@gmail.com (P.K.)

[2] Center of Nanostructures and Nanodevices (COINN), Universiti Teknologi PETRONAS, Seri Iskandar 32610, Perak, Malaysia; zainalarif.burhanudin@utp.edu.my (Z.A.B.)

[3] School of Electrical and Electronic Engineering, The University of Adelaide, Adelaide 5005, Australia, nelson.tansu@adelaide.edu.au

[4] Center for Photonics and Nanoelectronics, Department of Electrical and Computer Engineering, Lehigh University, 7 Asa Drive, Bethlehem, PA 18015, USA

* Correspondence: huzeinfahmi.hawari@utp.edu.my (H.F.H); monika_18000995@utp.edu.my (M.G.)

Dispersion stability

Figure S1 shows the optical photographs of the glass vials containing GO (A) and rGO suspension AArGO25 (B), AArGO50 (C), AArGO100 (D). It was noticed that after one day (24 h) of the storage, the AArGO100 began to agglomerate, indicating a higher degree of reduction. After two days, the sample AArGO100 was about to settle. This reflects the AArGO100 sample contains more basal plane functionalities [1]. However, AArGO25 and AArGO50 did not show any agglomerations and remained stable due to a comparatively low reduction of OFGs. On the fifth day, the sample AArGO100 was observed to be settled completely and sample AArGO50 just started to agglomerate whereas the sample AArGO25 remained stable. After ten days of storage, the samples AArGO50 and AArGO100 were found to be fully settled. On the contrary, the dispersion of sample AArGO25 was remained stable even up to one month, indicating to slight hydrophilic nature of the AArGO25 sample. The dispersion stability can be controlled by modifying the surface functionalities at the edges and the basal plane of rGO [2]. The presence of OFGs plays a crucial role in the dispersion stabilization of all rGO samples. The dispersion of edge functionalized graphene is better than basal plane functionalized graphene. Carbonyl and carboxyl OFGs contributes to better dispersion properties of the material [3]. Therefore, the removal of OFGs is supported by the agglomeration of rGO flakes due to π-π interaction between the layers.

It has been observed that the agglomeration rate rises with the concentration of ascorbic acid due to the high degree of reduction of OFGs. After 24 hours the rate of agglomeration rises from AArGO25 to AArGO100. Therefore, AArGO100 shows nearly complete elimination of oxygen-containing groups and has a larger area of the graphene network (see the presence of related peaks in FTIR spectra). The result suggests as the amount of ascorbic acid is increased, the degree of reduction is also increased. As a result, the highly reduced GO has a high tendency to agglomerate, and partially reduced GO has less tendency to agglomerate and shows high stability which was observed for the AArGO25 sample.

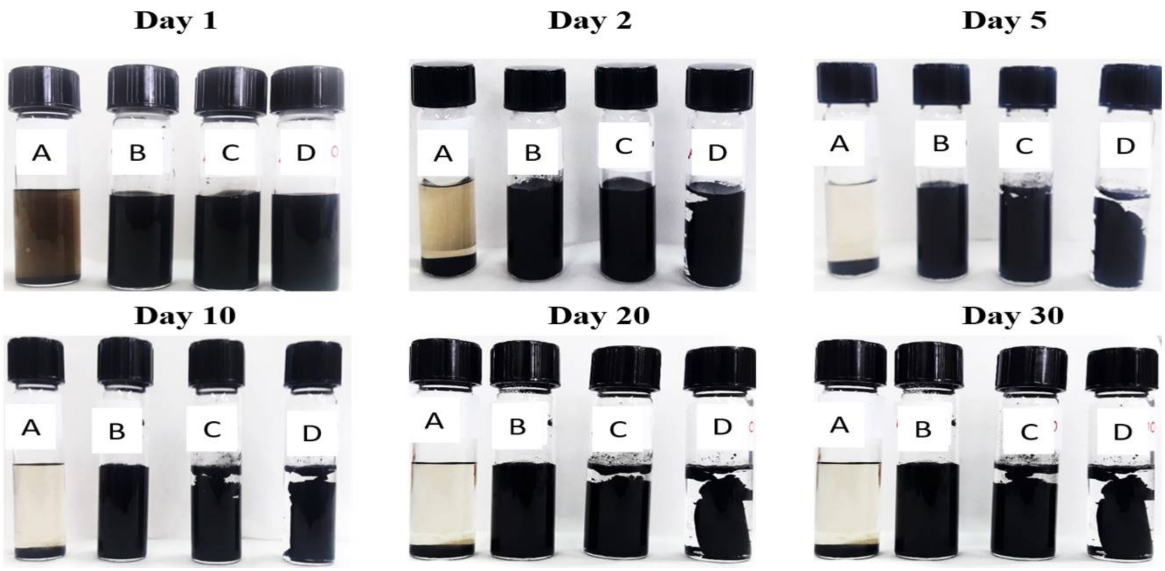

Figure S1. Aqueous dispersion of different AArGO: GO (A), AArGO25 (B), AArGO50 (C), and AArGO100 (D) after 1, 2, 5, 10, 20, and 30 days.

Table S1. Physical parameters of GO and AArGO25 obtained from XRD.

Sample	2θ(°)	d (Å)	cos θ	D (Å)	L(Å)	N	Miller indices	Crystal
GO	10.8	8.18	0.995	96	199.0	12	001	GO
AArGO25	21.8	4.07	0.981	5.9	12.25	04	002	rGO

References

1. Stankovich, S.; Dikin, D.A.; Dommett, G.H.B.; Kohlhaas, K.M.; Zimney, E.J.; Stach, E.A.; Piner, R.D.; Nguyen, S.T.; Ruoff, R.S. Graphene-based composite materials. **2006**, *442*.

2. Shih, C.J.; Lin, S.; Sharma, R.; Strano, M.S.; Blankschtein, D. Understanding the pH-dependent behavior of graphene oxide aqueous solutions: A comparative experimental and molecular dynamics simulation study. *Langmuir* **2012**, *28*, 235–241.

3. Chen, C.; Chen, Y.C.; Hong, Y.T.; Lee, T.W.; Huang, J.F. Facile fabrication of ascorbic acid reduced graphene oxide-modified electrodes toward electroanalytical determination of sulfamethoxazole in aqueous environments. *Chem. Eng. J.* **2018**, *352*, 188–197.
